# Cultural processes and demography: implications for conservation and beyond

**DOI:** 10.1098/rstb.2024.0145

**Published:** 2025-05-01

**Authors:** Philippa Brakes, Sasha R. X. Dall, Stuart Townley

**Affiliations:** ^1^ Centre for Ecology and Conservation, Biosciences, University of Exeter Faculty of Environment Science and Economy, Penryn TR10 9FE, UK; ^2^ Cetacean Ecology Research Group, School of Natural Sciences, Massey University, Auckland 0632, New Zealand; ^3^ Centre for Environmental Mathematics, University of Exeter Faculty of Environment Science and Economy, Penryn TR10 9FE, UK

**Keywords:** social learning, animal culture, conservation, population dynamics, culturally significant unit, learning biases

## Abstract

Social transmission of cultural variants in wildlife can cause population level effects with implications for conservation science, policy and practice. Social learning and animal culture can generate resilience in populations through the spread of adaptive behaviour but may also generate vulnerabilities. Distilling comprehensive management advice in this field remains challenging. Animal culture is important for defining ‘units to conserve’, managing human–wildlife interactions, reintroductions or translocations, and influences evolutionary change. However, the population level effects of cultural processes remain poorly understood. Given the breadth of issues for which cultural processes inform conservation, it is timely to consider the underlying processes in more detail. We consider the coupling of cultural processes and population dynamics to explore the conditions under which social learning can tip a declining population into growth. Simulations on a model system of two interacting cultural units are used to explore the tensions between the coupled dynamics of cultural and demographic processes. We show that even under a simple learning bias, the population level outcomes are complex. In concert with urgent targeted conservation action, we highlight the need to develop deeper process-based understanding in this field, to yield fundamental principles applicable to a broader range of encultured species.

This article is part of the theme issue ‘Animal culture: conservation in a changing world’.

## 1. Introduction

The emergent evidence for non-human (hereafter ‘animal’) cultures [1,2] has important consequences for conservation [3–5]. Culture and the social learning processes that drive culture can be important for how conservation is conducted (e.g. in managing human–wildlife interactions [6] and reintroductions [7]). Cultural processes can operate on both inter- and intra-generation timescales and can provide insights on how to delineate populations for conservation [8,9]. Here, we argue that the adaptive nature of cultural variants may be important when considering the implications for conservation. This is particularly the case for cultural variants that confer individual fitness benefits for survival or reproduction [10]. Such benefits may provide a useful target for conservation intervention. However, these systems are complex. The propagation of socially learned information is dependent on many factors, including the social learning process [11], transmission rates and substrate [12,13]. Therefore, clear advice on how to incorporate social learning and culture into conservation action remains elusive. While the role of social structure and social networks have been explored [12–14], how cultural processes interact with population dynamics remains poorly understood. The dynamics and reciprocity of cultural processes can generate patterning of importance to conservation [15,16] and feedback loops [17] relevant to conservation efforts [18,19].

As this field currently lacks fundamental principles on the interplay between cultural transmission and population dynamics, it is challenging to assess how applicable different approaches are across different biological systems and conservation contexts (e.g. [20,21]). There are many factors in play that can influence material outcomes. While there is good evidence that in certain circumstances culture can be a useful tool for delineating cetacean populations [9,16], applying this approach to other taxonomic groups remains challenging. For example, in chimpanzees, the granularity of the vast suite of evidence for cultural diversity both within and between chimpanzee groups and populations [22,23] may confound the idea of using a single cultural trait to delineate their conservation and these populations may be better served by conserving their cultural diversity [21]. But is this contrast in approach simply a matter of evidence base, or a difference in conservation setting? Is there something fundamentally different about how socially learnt behaviour is transmitted across these systems and is interfacing with population dynamics and conservation interventions? What does this mean for the management of fishes, birds or other mammalian populations? How might we better understand the underlying processes to identify the role of key individuals (see [6,14,24])? As our community develops pathways from the principle of conserving animal culture to understanding what this means in practice, we explore some of these endogenous processes, examine some of these questions and provide some first insights on thresholds and key factors that conservation scientists, policymakers and practitioners could consider when assessing the role of culture in conservation outcomes.

Participants of work conducted in this area over the last decade under the United Nations Environment Programme, Convention on the Conservation of Migratory Species (CMS), agreed on broad practical working definitions for culture and social learning, to encompass the suite of behavioural variation that has significance for conservation. Social learning is defined as any learning process facilitated by the observation of, or interaction with, another animal or its products [25–27]. Culture is defined as information or behaviours shared within a group and acquired from conspecifics through some form of social learning [26,28]. We adopt these definitions here.

From a conservation perspective, the central questions are:

(A) How does social learning facilitate the spread of adaptive behaviour to improve the resilience of wild populations facing intra- or inter-generational environmental or ecological challenges (e.g. [10])?
(B) How do specializations [16], or the social learning of less adaptive—or even maladaptive—behaviour [29,30], generate vulnerabilities in wild populations?
(C) How can the processes of social learning be harnessed to improve conservation interventions?

## 2. Culture, conservation and population dynamics

How social learning spreads across a network [31–34], how culture evolves [35,36] and influences genes through gene–culture coevolution [37] have been the focus of a great deal of research in the last few decades [2]. Nevertheless, how cultural processes scale up to have population-level effects is not well understood. Brakes & Whitehead [19] highlight some significant questions that remain for implementation in this field. These include whether managers should focus on specific content, social networks, variants that generate vulnerability, small culturally discrete units, or on the adaptive potential of specific variants [19].

Arguably, where one lands in answer to some of these questions is not just a matter of biology, but also philosophy. In addition to the functional value of culture to conservation efforts, there is a strong case to be made for the intrinsic value of non-human cultures (explored in [38]). Nevertheless, here we take a process-based approach to understanding the interplay between cultural transmission and population dynamics in the hope of shedding light on some of these key questions.

Examining the interplay between culture and demographic process in human populations, previous theoretical research demonstrated the value of exploring demographic structure and cultural transmission in concert, particularly for explaining domain-specific cultural behaviour as it changes over an individual’s lifetime and in shaping patterns of cultural evolution [39]. Similarly, in examining connectivity between human populations, simulations suggest that interactions between populations not only facilitate cultural transmission but may also generate a positive-feedback loop that can drive acceleration in cultural accumulation [17].

It is demonstrably the case that cumulative culture in the form of technology has increased the survival and reproduction of humans, from the agricultural revolution to genetic research [40,41]. While evidence for cumulative culture in other species is still emerging [42,43], there is good evidence that socially mediated behaviour, including social learning, can influence survival and reproduction in other taxa [44–56].

Exploring the implications of culture for conservation, a practical starting point is the influence of social learning on survival and reproduction [4], and how this may affect population resilience or vulnerability. Social learning can accelerate adaptive processes in populations and behaviours that enhance survival and reproduction and can spread more rapidly through social learning than through genetic evolution alone [8,9]. Maladaptive behaviours can also spread through social learning, especially if those behaviours are popular (frequency bias) or are demonstrated by highly influential individuals (prestige bias) [41]. Using population simulations, we examine here how a simple social learning bias can tip a population from decline into growth and demonstrate some of the nuance required to bring about such a tipping point.

## 3. Social learning bias and optimal thresholds for population growth

**Figure 1.**
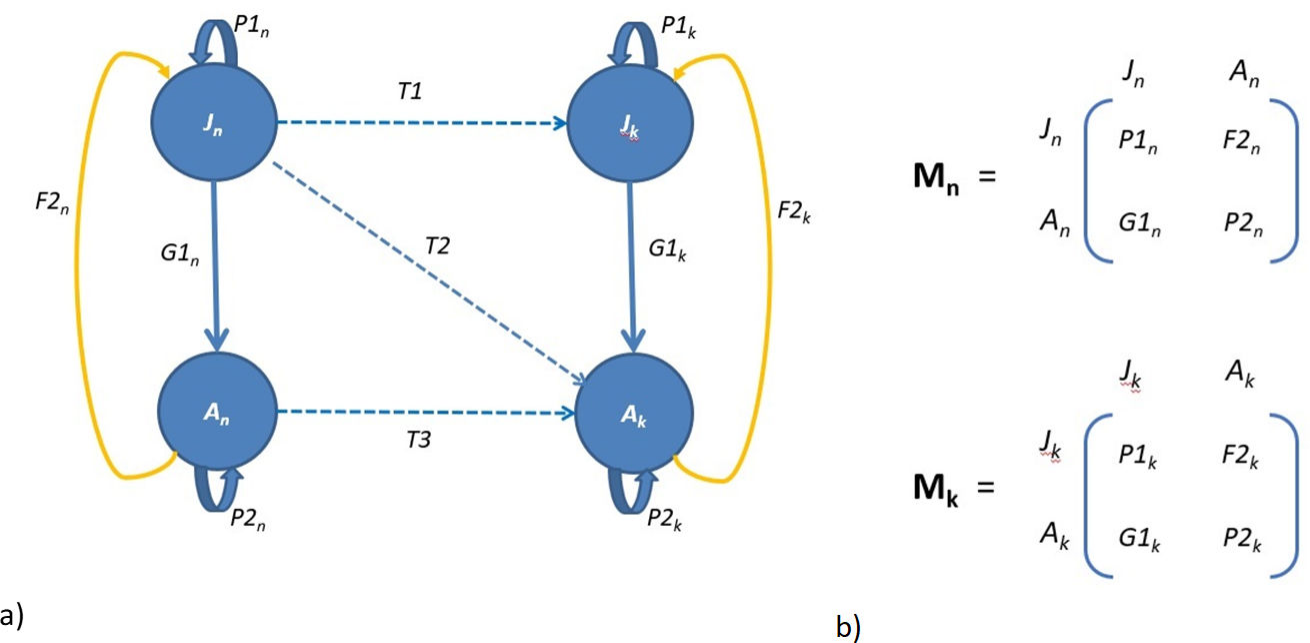
(a) Life cycle graph for two cultural units (*x*_*n*_ and *x_k_,* as described in the main text), where juveniles and adults can move between knowledge states, from naive to encultured (e.g. *J*_n_ to *J*_k_). Individuals survive with probability *P1_n_* or *P1_k_* (juveniles) and *P2_n_* or *P2_k_* (adults), move between stages with probability *G1_n_* or *G1_k_* and reproduce with fertility *F2_n_* or *F2_k_*. The transmission can be facilitated via transmission routes *T1*, *T2* or *T3. T1* represents horizontal transmission between juveniles, or vertical or oblique transmission from encultured adults; *T2* represents vertical or oblique transmission from encultured adults, or horizontal transmission from encultured juveniles, which results in naive juveniles transitioning to encultured adults; and *T3* represents horizontal transmission between adults. Note that in the wild, the movement of naive juveniles to encultured juveniles (i.e. *J_n_* to *J_k_*) may be the result of cultural transmission via *T1* and/or *T2* (i.e. horizontal and/or vertical or oblique transmission). (b) Population projection matrices for cultural units *x_n_* and *x_k_* with transition between stages as identified in (a).

While learning bias can influence the long-term evolution of cultures [57,58], there is a need to better understand the role of social learning biases in conservation outcomes [59] and in asymptotic and transient population dynamics. Using simulations of a model system of two interacting cultural units, we investigate the tension between the simultaneous dynamics of cultural transmission and demographic processes. A stage-structured population with two discrete cultural units (figure 1) is explored, to provide insights on how a directly biased social learning strategy (or content bias [60]) can influence concurrent demographic processes.

Consider two coupled, two-stage cultural units: *x*_*n*_, the naive cultural unit and *x*_*k*_, the encultured unit. Both units comprise juveniles (*J*) and adults (*A*) thus,


$$x_{n}=⟮\frac{J_{n}}{A_{n}}⟯\;\;x_{k}=⟮\frac{J_{k}}{A_{k}}⟯.$$

The population biology of the naive unit is determined by the population projection matrix (PPM) **M**_**n**_ and the encultured unit by **M**_**k**_ (figure 1b). These theoretical cultural units are sympatric and are linked through the movement of naive individuals (*x*_*n*_) to the encultured unit (*x*_*k*_) as the result of social learning of a novel foraging strategy (figure 1a).

The acquisition of variants is captured by transmission rates between naive and encultured cohorts and these can be manipulated depending on the transmission pathways to be modelled. At each time step, social learning can result in juveniles and/or adults moving from the naive unit (*x*_*n*_) to the encultured unit (*x*_*k*_), depending on the transmission route (*T*). The simple model of directly biased transmission of a dichotomous trait (or content bias) after Boyd & Richerson [61], provides for two cultural variants *n* and *k*, which can be considered as cultural ‘parents’. Both variants have equal weight in the transmission process, with parameter *B* representing the effect of directly biased transmission (where 0 ≤ *B* ≤ 1). The frequency of variant *k* before transmission is *p* and the frequency following transmission (*p′*) is calculated by the equation [61]


(3.1) $$p^{\prime }=p+Bp(1-p)\;.$$

Parameter *B* shapes the transmission of cultural variant *k*. For example, *B* = 0.9 would represent a very high transmission rate, in which the majority of the cultural unit uptake the new cultural variant over a short period. This might be comparable to the uptake of a novel foraging strategy transmitted under environmental constraints, such as a shortage of prey. Boyd & Richerson [61] suggest that equation (3.1) can be considered a model of vertical transmission but given the concept of ‘cultural parents’, it is argued here that this type of biased transmission can also be applied to horizontal or oblique transmission. This transmission function assumes that the overall population for each stage remains constant following transmission, as individuals are reallocated between variants. This function is inserted into our cultural population model such that cultural transmission takes place after population projection (i.e. *J*_*n*_ + *J*_*k*_ and *A*_*n*_ + *A*_*k*_ are the same before and after transmission). The transmission process then updates the demographics of the cultural units in each time step (electronic supplementary material, figure S1), with acknowledged parsimonious model assumptions (electronic supplementary material, table S1).

Under this scenario of transmission, with two PPM models coupled through simple content biased transmission, the key driver for propagation of the cultural variant of interest is the interaction between the proportion (*p*) of the focal cultural variant (in this case encultured individuals in *x_k_*), and the demography captured by the PPM. This can be written as


$$\begin{array}{l} x_{n}(t)\;{\;}^{\underset{\to }{demography}}\;\;x_{n}^{-}(t+1)\;{\;}^{\underset{\to }{transmission}}\;\;x_{n}^{+}(t+1)\;{\;}^{\underset{\to }{update}}\;\;x_{n}(t+1)\\ x_{k}(t)\;{\;}^{\underset{\to }{\;\;\;\;\;\;\;\;\;\;\;\;\;\;\;\;\;\;\;\;\;\;\;\;\;\;\;\;\;\;\;\;\;\;}}\;\;x_{k}^{-}(t+1)\;{\;}^{\underset{\to }{\;\;\;\;\;\;\;\;\;\;\;\;\;\;\;\;\;\;\;\;\;\;\;\;\;\;\;\;\;\;\;\;\;\;}}\;\;x_{k}^{+}(t+1)\;{\;}^{\underset{\to }{\;\;\;\;\;\;\;\;\;\;\;\;\;\;\;\;\;\;\;\;}}\;\;x_{k}(t+1). \end{array}$$

A key question from a management perspective is under what conditions might this coupled system ‘flip’ from decline into growth, or vice versa, and how might understanding the dynamics and optimum thresholds generated inform the conservation of species that learn socially (see questions A and B, §1).

**Figure 2.**
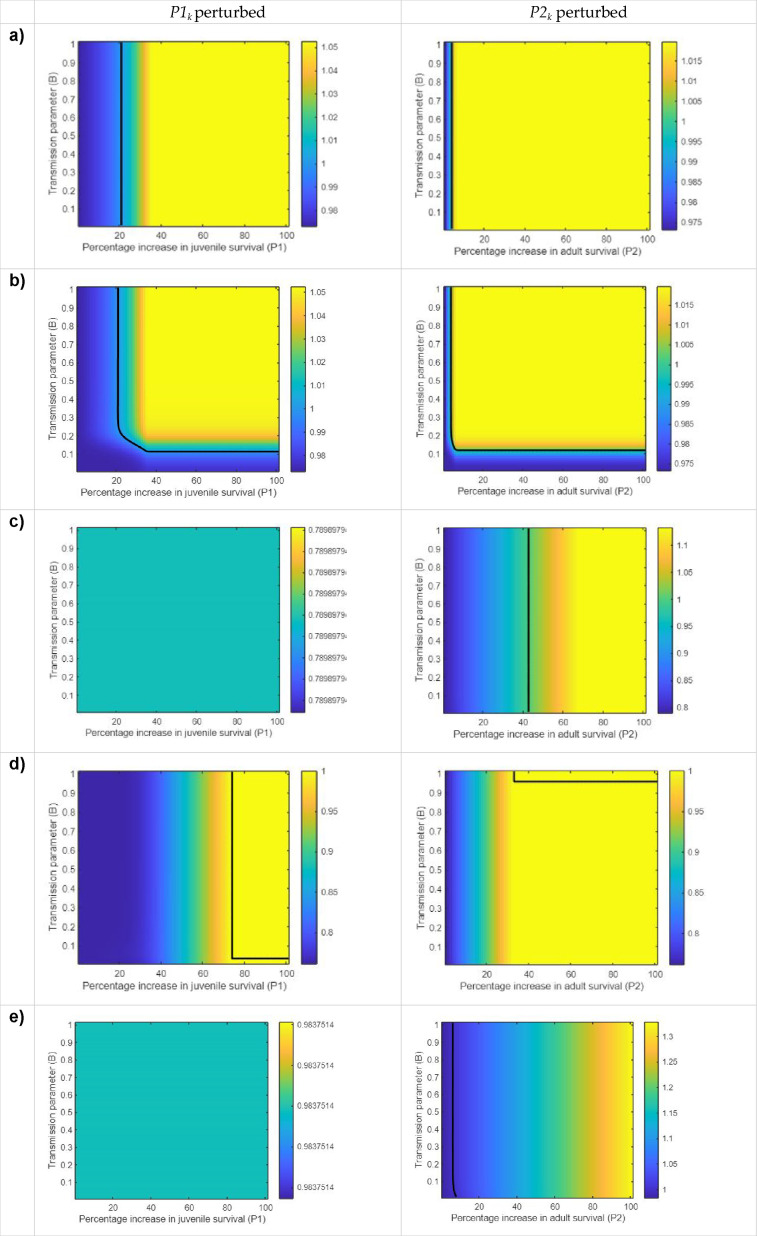
Parameter space exploration for an incremental increase in the survival rate of juveniles (*P1_k_*) and adults (*P2_k_*) following horizontal transmission between adults through directly biased social learning. The colour bar represents the growth rate (*λ*). The black contour line indicates the threshold where the combination of incremental change in transmission (*B*), coupled with the percentage increase in survival, results in a stable population, where *λ* = 1. Simulations were undertaken using PPM data from the COMADRE database (see the electronic supplementary material) for sperm whales (a,b), Douglas squirrels (c), green-rumped parrots (d) and barn swallows (e). In all cases, except scenario (b), the naive and encultured units were initially equal (*x*_*n*_ = *x*_*k*_), where *J_n_* = 10, *A_n_* = 10, *J_k_* = 10 and *A_k_* = 10. In scenario (b), the naive unit was initially significantly larger than the encultured unit, with *J_n_* = 400, *A_n_* = 8000, *J_k_* = 4 and *A_k_* = 10, to explore the effects of relative abundance.

To examine these coupled dynamics, a series of simulations were undertaken to explore the combined effects of horizontal cultural transmission and population dynamics. Parameterizing the model with vital rates for a selection of declining mammal and bird populations to explore species with different life histories, parameter space explorations were undertaken for 0.01 incremental increases in survival rate, in concert with 0.01 incremental increases in the transmission parameter (*B* = 0 to 1), iterated 100 times. Species were selected from the COMADRE database on the basis of published 2 × 2 PPM data from declining species with a range of life histories (see electronic supplementary material, table S2). The simulations were undertaken for a range of initial conditions, to explore the optimal threshold between the coupled dynamics of the increase in transmission parameter (*B*) and the improvement in survival of the encultured unit, (*P1*_*k*_ and *P2*_*k*_), to bring about growth in the overall population (i.e. *λ* > 1). A contour line was fitted in each simulation, where *λ* = 1, to identify the threshold at which the previously declining overall population (*x*_*n*_ + *x*_*k*_) becomes stable and beyond which (λ > 1), the population transitions into growth (figure 2).

We might intuitively anticipate that the most important factors governing outcomes in such a system is either the improvement in survival rate or the ratio of encultured to naive individuals in the population, relative to the increase in survival. However, these simulations show a range of outcomes (figure 2), where the feedback between improvement in vital rate and shared culture can tip this system into growth. The relative abundance of the naive to encultured units in the initial population appears to play a role in determining the threshold for the combination of the transmission and the vital rate parameters (here survival), to bring about growth (see electronic supplementary material, figure S2a,b). This may be the result of an initial population with more naive individuals than encultured, i.e. the system must pass a threshold of transmission before the benefits of the contribution of transmission and survival increment together shift the population into growth. But stage structure (the relative abundance of juveniles to adults) may have a significant role in determining the threshold for growth in this system.

In some simulations, there is no tipping point to population growth, despite a 100% increase in survival (figure 2c,e). For example, even a 100% increase in juvenile survival rate in the Douglas squirrel (*Tamiasciurus douglasii*) simulation (figure 2c), did not tip the system into growth. Between adults, transmission is sufficient, when coupled with an incremental increase in juvenile or adult survival in other scenarios to eventually bring about growth (e.g. green-rumped parrot, *Forpus passerinus*, figure 2d). These results are somewhat counter-intuitive and warrant future exploration as they are likely the result of the coupled dynamics of this system but may also relate to the life history of the species, as expressed through the PPM parameters (electronic supplementary material, table S2).

Here, it has been shown that a cultural variant with rapid transmission (*B* = 0.9), e.g. a cultural fad that spreads quickly through a population, has a very different impact on population dynamics than the spread of a cultural variant with a much lower transmission rate (*B* = 0.1). The relative abundance of cultural units, as well as stage structure, can also influence how socially learnt behaviour propagates. This system does not include any density dependence and limits to growth, which are inherent in real-world systems. Here, we have focused on culturally transmitted behaviour that is adaptive, but such constraints could also apply to the transmission of maladaptive cultural variants. For example, if climate shifts and cultural traits no longer track local conditions.

## 4. Discussion

The central proposition of this article is that animal culture can influence population dynamics to generate population level patterns and processes that in turn may inform conservation science, policy and practice. Even in the relatively simple model system presented here, the coupling of stage-structured population dynamics with a simple form of social learning can have a range of effects, depending on the parameter regime. The strength of transmission can influence not only the movement towards the encultured population in this system (and thus the outcomes for both cultural units) but also how the benefits to adult survival or reproduction of a cultural variant impacts population dynamics (see question C, §1). It is notable that these effects can sometimes be demonstrated with a less than 10% increase in adult survival (e.g. barn swallow, *Hirundo rustica*, figure 2e). This potentially provides an opportunity in some conservation settings, such as translocations, to manipulate transmission parameters to capitalize on marginal gains in survival or reproduction for the benefit of a wider population.

The parameter regimes required to ‘tip’ a population into growth (or decline) in our model are very specific and contingent on a number of variables. The outcomes from the simple system examined here depend on a complex interplay between improvement in vital rate resulting from the cultural variant and the rate at which the variant is transmitted. The optimal parameter regime for growth (λ ≥ 1), as well as being dependent on the transmission parameter (*B*) and incremental change in survival, is also contingent on the relative abundance of the naive (*x_n_*) and encultured (*x_k_*) units in the initial population (see figure 2a,b). Further, the complex outcomes in these simulations indicate that beyond influencing asymptotic dynamics, there may be shorter term transient dynamics under certain parameter regimes, where the system moves between equilibria. Exploring these dynamics may be particularly relevant for responses to rapid environmental change and the timing of conservation intervention, potentially providing early warning signals, e.g. where a temporary rapid increase in abundance may signal an oncoming decline.

### (a) Social learning, culture and population assessments

In §1, we highlighted some key questions on the implications of social learning for generating both resilience and vulnerability and how these processes may be harnessed to improve conservation efficiency. A key objective of conservation is to maintain heritable variation and adaptive evolvability [2]. The aim of conservation policymakers is to determine the most salient unit to conserve to achieve the best conservation or sustainability outcomes, but the processes of social learning can complicate assessments. Delineating populations is a primary consideration and can be informed by data on genetics, population structure, movement between populations and non-human culture [4,9]. Such data enable the estimation of abundance and trends that can inform threat status or sustainability [62]. An important element— particularly the case for cetaceans given the challenges associated with accurate abundance estimates for these species [63]—is estimating the degree of uncertainty associated with these estimates [64]. To calculate uncertainty, managers must consider a wide range of factors that include endogenous population processes and the exogenous influence of the environment affecting population dynamics. Given the evidence that non-human culture can generate both heritable variation and cultural structuring of populations [2,8,9], we have shown here how social learning, when coupled with the processes of population dynamics, can generate complex and unexpected outcomes. Therefore, social learning is arguably an important endogenous process for evaluating uncertainty in population assessments.

The concept of the evolutionary significant unit (ESU) was developed in response to the need to establish units that conservation managers and practitioners should aim to conserve [65,66]. ESUs focus specifically on genetic or heritable phenotypic distinctiveness, within units that demonstrate isolation, such that there is a restricted flow of information that determines genotypes or phenotypes, from other such units [65,66]. Whereas, a demographically independent population (DIP) is focused specifically on internal demographic processes (births and deaths) that are more important to population persistence than migration [67,68]. In addition, a cultural variant is a particular form or variant of a cultural trait displayed by a group or population [69]. It has since been argued that after genetics, culture may be the second most important process for shaping phenotypic diversity within and between populations [26]. Since social learning can create heterogeneity, which can result in the cultural structuring of populations that can be differentially impacted by anthropogenic threats [23], it has been demonstrated here that culturally structured populations can generate unexpected outcomes, which may inform the ‘unit to conserve’ [9]. The question then for conservation managers is what are the risks involved in incorrectly assuming that all populations are culturally homogeneous? Given the complex dynamics between culture and population dynamics explored here, if managers attempt to incorporate culture into conservation assessments, what criteria should they use to decide which units to prioritise?

### (b) Identifying key factors

The complex dynamics between animal culture and conservation [8,66,70] have been considered from a number of perspectives: from exploration of the role of individuals and social networks [14], or how culture can delineate populations [9], to responses to anthropogenic threats [71] or the use of agent-based simulations to explore how innovations spread and generate conformity under different social learning rules [72]. Nevertheless, distilling central questions underlying the application of culture to conservation has taken some time [19]. Beyond the intrinsic value of cultures (see [38]), a key issue for managers is how to support adaptive cultural variants that confer fitness benefits, as these can provide a compelling target for conservation intervention. It may be possible to achieve this by maintaining existing cultural diversity [21], but this must be supported by efforts to conserve cultural capacity across populations [4]. Cultural capacity can be maintained by supporting the biotic and abiotic conditions for adaptive variants to arise [38], or by supporting the conditions for cultural transmission of behaviour such as bird song [56] and whale song [73]. While cultural ‘content’ can be important (e.g. a variant’s potential adaptive value), the patterning and dynamics that this content generates are also highly relevant. Returning to our original ‘central questions’ (§1), the grand challenge [74] for the field of culture conservation is identifying principles that can be applied across multiple systems, with combined insights from theoretical and empirical ecologists.

The exploration of the different parameter regimes presented here demonstrates the complex interplay between different aspects of this system, even under a scenario with a relatively simple social learning rule. Our simulations support the argument that social learning biases should be taken into consideration for reintroductions and translocations (see ‘Conservation application’ below) [59]. Moreover, these simulations indicate that social learning biases can be important for factors as diverse as decision-making on delineating cultural units, to issues such as managing human–wildlife interactions [6] and cultural rescue [75].

### (c) Conservation application

For some cetacean species, cultural behaviour has been successfully implemented in policy as a delineator, where culturally transmitted foraging strategies or migration influence habitat use or survival [76–78]. The Committee on the Status of Endangered Wildlife in Canada (COSEWIC) lists Southern Resident killer whales (*Orcinus ater*) (SRKW) in the Pacific Northwest [79] and beluga (*Delphinapterus leucas*) that summer in eastern and western Hudson Bay [80], as distinct units on the basis of culturally transmitted behaviour (SRKW are also listed as a distinct population segment under the Endangered Species Act in the USA [81]). In addition, southern right whales (*Eubalaena australis*) exhibit culturally transmitted, maternally led site fidelity and have been recognized as ‘DIPs’ in New Zealand and Australia and through IWC stock assessments [76,82].

Nevertheless, there are challenges in moving from theory to practice in this field. While there has been progress in using culture to delineate cultural units in some cetacean species [9,19,83], culture has rarely been used explicitly to delineate conservation units in other taxonomic groups. This may be, in part, due to the challenges of unravelling the complex processes in play. In sperm whales (*Physeter macrocephalus*), the fact that acoustic codas are vertically transmitted and are relatively stable, generating distinct measurable patterning between groups, which may relate to habitat use or foraging strategies [84] (see [20,85]), make culture a viable delineator [9]; particularly where there may be fitness costs or benefits [47]. Factors for identifying culturally distinct units within a wider population include the discreteness and stability of a cultural variant and whether the variant directly influences survival, reproduction or dispersal. But there are arguably other important factors, such as population and social structure, transmission pathway, social learning biases, fidelity of copying and how cultural transmission relates to the use of space. Moreover, in some instances, conserving broader cultural capacity may be the more salient conservation target. While understanding the complexity of these systems is in its infancy, the key objective moving forward is to resolve some of this complexity to identify instances where culture is a useful metric for informing conservation management options. However, beyond the underlying biological processes, the reality of the conservation landscape and the politics of conserving different species also have a crucial role [21].

Returning to the question of how social learning can be harnessed to improve conservation interventions (§1, question C), these simulations show how the relative abundance of encultured individuals can influence wider population dynamics. Understanding the interplay between the parameters in these systems may be important during interventions such as reintroductions, where the relative abundance of encultured to naive individuals can influence success as survival strategies are shared (e.g. [86–88]) and may inform the timing of interventions. Stage structure, i.e. the relative abundance of juveniles to adults in each cultural unit, and overall abundance (*x_n_* + *x_k_*) of the wider population may also be highly relevant (e.g. to prevent the acceleration of the Allee effect [89]). In different conservation settings, managers may more easily be able to manipulate transmission rates (e.g. through the provisioning of materials for nest sites, or encultured demonstrators in golden lion tamarins (*Leontopithecus rosalia*) [86]), or survival rates (e.g. by reducing threats). These simulations provide preliminary insights on additional factors that may be relevant for lasting outcomes.

Life history parameters, such as time spent by an organism in a specific developmental stage and probability of surviving (and/or reproducing) and ageing into the next stage, are also important considerations for how cultural information is transmitted across a population. If cultural transmission of a specific foraging strategy results in increased survival in reproductive adults (*P2*) within a cultural unit, these encultured adults may ‘boost’ the population by continuing to reproduce for longer, with the added benefit (positive feedback) of continuing to transmit the skills associated with the advantageous foraging strategy. In contrast, the selective removal of older, encultured cohorts could have a more disproportionate effect than the initial reduction in census size (see [88]).

To grow understanding in this field, our results suggest that managers should be encouraged to incorporate some of the following into conservation assessments for species that learn socially:

(i) explore the temporal and spatial patterning created by cultural transmission and examine effects on survival, reproduction, dispersal or habitat use;
(ii) gather data—or reexamine existing data using a cultural lens—to explore the propagation of cultural variants across social networks and structures, and evaluate the relative abundance of encultured to naive units and individuals, to help elucidate the thresholds for transmission and vital rate parameters to bring about growth; and
(iii) where possible, determine the route of social transmission (horizontal, vertical or oblique) and whether the variant is ubiquitous across the population, or in isolated or sympatric cultural units.

Our results demonstrate that despite the daunting complexity, applying social learning theory to population dynamics can help identify populations or cultural units that are likely more resilient or vulnerable, enabling more targeted interventions that foster the transmission of adaptive cultural variants. While it is essential to balance pressing conservation issues and limited resources for conservation, it would be short-sighted not to include theoretical research in helping to drive the frontier of this field and edge us closer to distilling salient management advice for some of the most critically endangered species. The outcomes for many species are heavily guided by management models (e.g. ‘potential biological removals’ [90]). However, these models usually fail to capture the type of complexity that can be generated by cultural transmission. Cultural processes can provide insights on vulnerability and can be harnessed to improve the efficiency of existing efforts, by capitalizing on the intra- and inter-generational effects of social learning. Theory does not operate in a vacuum. We urge collaboration between theoretical and empirical ecologists to advance this field [74]. But this must be carefully balanced with the need for urgent conservation action for so many endangered species. We cannot defer action while we wait for data or insights that are currently out of reach.

## 5. Concluding remarks

Many populations are in urgent need of conservation action. It is imperative to balance mitigating threats and urging the relevant actors to take action, with the desire to develop a deeper understanding of the underlying processes. We argue that there is a need to develop fundamental principles in this field to better predict longer term outcomes and improve conservation efficiencies.

These simulations suggest that there are many factors that influence how the processes of social learning can impact conservation, including the nature of the substrate upon which cultural variants act. This research also clearly points to the need for more nuanced definitions of *what* managers should be aiming to conserve, by protecting social learning pathways across social networks, within and between populations. A deeper understanding of the interactions between cultural processes and demography may also be particularly helpful for informing *how* conservation is undertaken, during interventions such as cultural rescue [75] or reintroductions [7]. This research highlights the need to better understand how learning biases [59] can influence practical aspects of conservation and how these processes can scale up to influence demography and impact populations in the longer term.

Animal culture presents an enormous challenge for conservation science, policy and practice, as well as an enormous opportunity. The systems and processes involved are complex, but the rewards of being able to harness insights on some of these processes for the benefit of biodiversity will be manifold. Acknowledging the inherent challenges of adapting culture theory into conservation practice, we argue that it is precisely because cultural transmission can influence ‘well established conservation metrics such as population size, viability and demography’ [91], to increase population resilience or create vulnerability, that theoretical research can provide insights for empirical studies to elucidate how best to harness some of these processes for conservation benefit.

While we have shown some of the interaction between cultural processes and population dynamics, many questions remain. Animal culture is undoubtedly a powerful tool for conservation advocacy, but to focus only on this aspect risks missing a behemoth of functional information flow across populations, in some instances moving more rapidly and effectively than the flow of genetic information (e.g. [31,48]). In the absence of fundamental principles in this field there is an urgent need to develop criteria for conserving cultural capacity within and between populations so that adaptive cultural variants can flourish [38]. It is only through a combination of theoretical and empirical research, as well as real-world implementation, that knowledge of animal culture can assist timely protection of biodiversity, providing insights on the conditions for populations and species to flourish.

## Data Availability

Simulation script and PPMs are provided in the electronic supplementary materials [92].
